# (Mal)adaptive Mentalizing in the Cognitive Hierarchy, and Its Link to Paranoia

**DOI:** 10.5334/cpsy.117

**Published:** 2024-09-11

**Authors:** Nitay Alon, Lion Schulz, Vaughan Bell, Michael Moutoussis, Peter Dayan, Joseph M. Barnby

**Affiliations:** 1 Department of Computer Science, The Hebrew University of Jerusalem, Jerusalem, Israel; 2 Department of Computational Neuroscience, Max Planck Institute for Biological Cybernetics, Tübingen, Germany; 3 Clinical, Educational, and Health Psychology, University College London, United Kingdom; 4 Department of Imaging Neuroscience, University College London, London, United Kingdom; 5 Department of Computer Science, University of Tübingen, Tübingen, Germany; 6 Department of Psychology, Royal Holloway University of London, London, United Kingdom; 7 School of Psychiatry and Clinical Neuroscience, The University of Western Australia, Australia

**Keywords:** Theory of mind, paranoid delusions, paranoia, emergence of paranoid behavioir, computational psychiatry, computational model, Bayesian brain

## Abstract

Humans need to be on their toes when interacting with competitive others to avoid being taken advantage of. Too much caution out of context can, however, be detrimental and produce false beliefs of intended harm. Here, we offer a formal account of this phenomenon through the lens of Theory of Mind. We simulate agents of different depths of mentalizing within a simple game theoretic paradigm and show how, if aligned well, deep recursive mentalization gives rise to both successful deception as well as reasonable skepticism. However, we also show that if a self is mentalizing too deeply – hyper-mentalizing – false beliefs arise that a partner is trying to trick them maliciously, resulting in a material loss to the self. Importantly, we show that this is only true when hypermentalizing agents believe observed actions are generated intentionally. This theory offers a potential cognitive mechanism for suspiciousness, paranoia, and conspiratorial ideation. Rather than a deficit in Theory of Mind, paranoia may arise from the application of overly strategic thinking to ingenuous behaviour.

**Author Summary:**

Interacting competitively requires vigilance to avoid deception. However, excessive caution can have adverse effects, stemming from false beliefs of intentional harm. So far there is no formal cognitive account of what may cause this suspiciousness. Here we present an examination of this phenomenon through the lens of Theory of Mind – the cognitive ability to consider the beliefs, intentions, and desires of others. By simulating interacting computer agents we illustrate how well-aligned agents can give rise to successful deception and justified skepticism. Crucially, we also reveal that overly cautious agents develop false beliefs that an ingenuous partner is attempting malicious trickery, leading to tangible losses. As well as formally defining a plausible mechanism for suspiciousness, paranoia, and conspiratorial thinking, our theory indicates that rather than a deficit in Theory of Mind, paranoia may involve an over-application of strategy to genuine behaviour.

## Introduction

To be strategic, and thus sometimes also deceptive, we need to take into account the beliefs, desires and intentions of others. The cognitive process underlying such behaviour is theory of mind (ToM) – an agent’s ability to reason about latent characteristics of others; what they know, want or plan (Dennett, 1989; Premack & Woodruff, 1978).

Signatures of ToM have captured the attention of computational scientists who have formalised ToM as a collection of social processes that enable inference and representation about the dynamic interaction between a self and other(s) (Barnby, Bellucci, et al., 2023; Ray, King-Casas, Montague, & Dayan, 2008; C. Baker, Saxe, & Tenenbaum, 2011; C. Baker & Tenenbaum, 2014; C. L. Baker, Jara-Ettinger, Saxe, & Tenenbaum, 2017; Goodman et al., 2006). At the most shallow level, an agent (’the self’) simply considers the utility function (the desires) or beliefs of another agent (the ‘other’) based on their past behaviour (Ng & Russell, 2000; Jara-Ettinger, 2019). This can be extended to deeper levels recursively: You can think about what I think you think I think (what you think, etc.). Hierarchical ToM – the ability to hold nested beliefs of ourselves and others (Camerer, Ho, & Chong, 2004; O’Grady, Kliesch, Smith, & Scott-Phillips, 2015) – has been suggested as supporting the way that humans choose what to say or teach to maximise interpretability (Goodman & Frank, 2016; Barnett, Griffiths, & Hawkins, 2022), and as underlying cognition in social, competitive settings (Devaine, Hollard, & Daunizeau, 2014a). It allows agents to hide information from others strategically, and to use an opponent’s inference process against them in forms of deception, skepticism, and strategies to overcome these (Alon, Schulz, Rosenschein, & Dayan, 2023; Doshi, Qu, & Goodie, 2014).

With ToM’s outsized role in human interaction (Devaine, Hollard, & Daunizeau, 2014b), it is unsurprising that failures of ToM have been suggested as being at least part of the basis of several psychiatric disorders (McLaren, Gallagher, Hopwood, & Sharp, 2022), such as autism (Frith & Happé, 1994; Yoshida et al., 2010; Chiu et al., 2008), psychosis (Bentall & Kinderman, 1998; Randall, Corcoran, Day, & Bentall, 2003; Penn, Sanna, & Roberts, 2008), and personality disorders (Sharp et al., 2011; Hula, Montague, & Dayan, 2015a; Galvez-Merlin et al., 2023; Rifkin-Zybutz et al., 2021; Euler et al., 2021; King-Casas et al., 2008).

In patients with persecutory delusions and those with high paranoia, there is a tendency to make personal, external attributions – that is, explaining the causes of negative events through the malicious intentions of others (Buck, Browne, Gagen, & Penn, 2023). In borderline personality disorder (BPD), individuals are theorised as attributing an excessively high level of intentionality to sparse social data (Sharp et al., 2011). Here, over-mentalizing or hyper-mentalizing is defined as “making excessively convoluted inferences based on others’ social cues” and (Fonagy, Luyten, & Bateman, 2015) has been suggested as giving rise to paranoia in BPD (McLaren, Gallagher, Hopwood, & Sharp, 2022), and was shown empirically to be related to early stages of disorder (Galvez-Merlin et al., 2023). In both psychosis and BPD, and even more commonly in conspiratorial ideation (Bowes, Costello, & Tasimi, 2023), there is a higher risk of *over*-interpreting behaviour as being more sophisticated, intentional, and malicious.

Nevertheless, the cognitive mechanisms of this approach to paranoia within persecutory delusions and BPD have been hard to pin down and specify with the dynamic interaction and representation of social agents making mechanisms harder to examine (Penn et al., 2008; Bell, Mills, Modinos, & Wilkinson, 2017). Traditionally ToM has been measured using vignette tasks. While these are typically predictive of psychosis (Fett et al., 2011), and to some extent persecutory delusions (Corcoran et al., 2007), a major limitation is that they do not take into consideration the self-representation of the participants, nor the representation of others about their sense of self (Chan & Chen, 2011). Some computational and experimental work using game theory paradigms suggests that high paranoia and psychosis are affiliated with rigid, slower-to-update priors about the self-relevant nature of a partner’s actions (Fett et al., 2012; Barnby, Mehta, & Moutoussis, 2022; Barnby, Bell, Deeley, Mehta, & Moutoussis, 2023). This begs the question as to whether ToM changes in paranoia and paranoia-affiliated diagnoses may be caused by changes in the maladaptive application of recursive cognition in social settings. There has been little work examining the role of cognitive recursion applied to BPD, paranoia, and persecutory delusions, aside from some notable exceptions (Hula, Vilares, Lohrenz, Dayan, & Montague, 2018; Hula et al., 2015a), which did not focus on false belief generation or maintenance.

Here, we offer an example of the ramifications of being adaptively and maladaptively strategic at different recursive levels. We use simulations based on Interactive Partially Observable Markov Decision Processes (IPOMDP) (Gmytrasiewicz & Doshi, 2005) to suggest how this can help explain social cognitive processes that result in paranoia, suspiciousness, and/or conspiratorial ideation. We show how the degree of reasoning about the intentions of others (Ho, Saxe, & Cushman, 2022; Premack & Woodruff, 1978; Devaine et al., 2014b) can be a protective factor against exploitation. However, we also demonstrate how this can go grossly awry: Selves that over-interpret actions of others make misplaced inferences about the others’ strategic and deceptive intentions, with a malign effect on the reward garnered by the self.

We begin by emphasizing the importance of hierarchical mentalizing in mixed-motive games. These sequential social dilemmas (SSD) serve as a tractable testbed to observe the emergence of complex behaviour (Alon, Schulz, Rosenschein, & Dayan, 2023), as agents need to balance their reputation with material gains and losses. This work reinforces previous findings showing that agents with deep mental recursion, known as their Depth of Mentalization (DoM) can successfully manipulate the beliefs of those one-step lower in the hierarchy (Alon, Schulz, Rosenschein, & Dayan, 2023; Alon, Schulz, Dayan, & Barnby, 2023).

Next, we present the potential downside associated with maladaptively high DoM, i.e., hyper- or over-mentalizing. This pitfall is illustrated through a sequence of interactions between agents with mismatched DoM. We show that agents with maladaptively high DoM overestimate the complexity of their counterparts and overreact to sincere agents. This overreaction yields detrimental results. We then discuss how these results have the potential to explain some key aspects of psychopathology.

**Definition: Overmentalizing**

When properly calibrated, Theory of Mind (ToM) is used to reason about the mental state of others, inferring their intentions from actions, and thus being able to respond or anticipate appropriately in the future. In formal terms has been framed as a hierarchical system: a DoM(*k*) agent properly models a DoM(*k* – 1) agent and utilizes this ability to predict and affect the behaviour of that DoM(*k* – 1) agent.

This fixed hierarchy assumes that a self can model others one step below. What has not been traditionally considered is what happens when we infer sophisticated or complex intentions to otherwise simple or ingenuous behaviour. To put this into context, imagine walking in the street and seeing a friend. You wave, but your friend does not wave back. The reality is that your friend has not seen you, but this is unknown to you. A simple interpretation is to assume they did not see you and therefore did not wave. A more recursive hypothesis is that your friend did see you, but chose not to wave. This interpretation assumes your friend has a more complex model of the situation, one that involves you and a conscious choice to ignore you. Lastly, one can imagine an even deeper recursion, one in which you assume that not only your (so called) friend saw you and decided to ignore you, but that wasn’t by accident that you two met on the street – rather they followed you to that street, planning to cause you to be upset by pretending to not see you. Formally, we can think of our friend as a DoM(*k* = –1) agent in this context – they are making (or omitting actions) without any regard to you. The first interpretation is an example of a DoM(*k* = 0) belief – one that is focused on the other with no regard to the self. The second interpretation uses a DoM(*k* = 1) model, which means that your friend is thinking about you at the time when they omitted to wave. Thus, as a general rule, when the observed agent’s DoM is lower than (*k* – 1), the DoM(*k*) agent overestimates the complexity of the observer’s world model, which leads to an overly sophisticated internal simulation of potential intentions that accounted for their actions. This, in essence, is overmentalizing.

Our work offers lessons to several fields: To the computational cognitive science, and psychiatry communities, we offer a computational account of a process contributing to paranoid beliefs and behaviour, and a possible mechanism underlying excessive recursive belief formation in general psychopathology. We show the AI community how ToM needs careful calibration to avoid counterproductive inference, and hence loss of veridicality and reward between agents. As a result, our work has key implications for AI safety and human-computer interaction.

## Materials and methods

**Figure 1:**
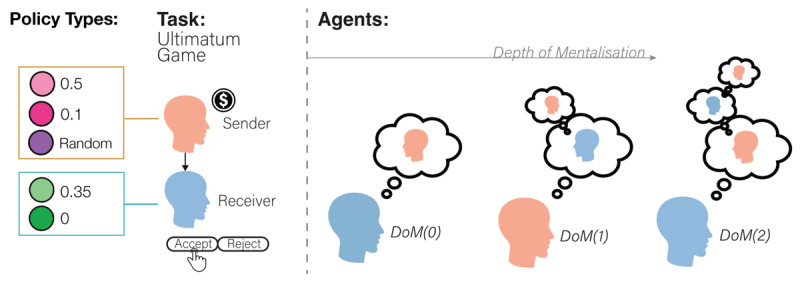
**Task and Agent Summary:** In the Ultimatum Game, a sender (orange) chooses how much of an endowment to send to a receiver (blue). The receiver then has a chance to either accept or reject this offer. If the receiver accepts, they both get to keep their portion of the endowment. If the receiver rejects, neither gets anything. In our simulations, we included two types of sender and two types of receiver. The first type of sender has a Depth of Mentalization of –1 (DoM(–1)) – it possesses no Theory of Mind and is simply reactive to the receiver’s actions. In addition, we introduce a random sender, sending uniformly distributed offers. The other type of sender and both receivers are endowed with Theory of Mind along with DoM \[
\in \left\{0,\,1,2\right\}
\]. This enables these agents to model their partners recursively, to a strictly limited extent. Both agents are characterized by their DoM level and by a threshold, representing in principle the minimal reward they are willing to accept. Agents at DoM(>0) can conceptualise the world model of how others below them in the hierarchy perceive the self. Given the requirement of the IUG to balance short and long-term rewards, and given the types of agents available, this leads to strategic play by more sophisticated agents to get a greater frequency of more favourable outcomes. For example, a DoM(1) agent knows that the DoM(0) is able to conceptualise whether they are playing with a random or intentional sender, and therefore may behave in a way that causes the DoM(0) to mischaracterise the sender’s identity as random. This means the DoM(1) can then send very unfavourable offers knowing that the DoM(0) does not believe they can influence the outcome.

Mixed motive games offer a particularly useful test bed to examine the rise of complex behaviour and test the role of opponent perception in social interactions. Generally speaking, a mixed-motive game is an interaction between two or more agents where there are competing or mixed preferences over the outcome. One such game is the Prisoner’s Dilemma, where both parties gain more from mutual cooperation than from mutual defection, while one side can gain an even higher reward by defecting from a cooperating partner. In this work, we match agents with increasing degrees of DoM in the Iterated Ultimatum game (Xiang, Lohrenz, & Montague, 2013; Alon, Schulz, Dayan, & Barnby, 2023) IUG (Figure 1). This game is comprised of *T* > 0 repetitions of the following game: a *sender, S*, is endowed with monetary units, set in this work to 1. They then offer the *receiver, R*, a partition of this endowment: the receiver would get *a_S_* while the sender would get to keep 1 – *a_s_* for themselves. The receiver then decides whether to accept the offer (*a_R_* = 1) or to reject it (*a_R_* = 0). In the latter case, both parties get zero reward. The structure of the utilities makes the IUG a mixed-motive game: the sender’s utility decreases with the offer size and so they are incentivised to offer the receiver less. However, if the sender offers too little, they will end up with nothing. Hence the sender has to balance their desires with those of the receiver to maximize their long-term utility.

We use the superscript *t* to denote the actions of both agents at trial \[
t\in \left[1,T\right]
\]: \[
a_{S}^{t}, a_{R}^{t}
\]. In turn, we define the *history* at time *t* as the sequence of offers and responses: \[
{{h}^{t}}=\langle a_{S}^{1}, a_{R}^{1}, \ldots, a_{S}^{t}, a_{R}^{t}\rangle
\].

Apart from a particularly simple, random, sender, each agent, \[
i\in \left[R, S\right]
\] is characterized by two parameters: its utility function: \[
{{u}_{S}}, {{u}_{R}}
\] and its DoM level: \[
k\in \left\{-1, 0, 1, 2\right\}
\]. The utility is governed by a threshold \[
{\eta}_{S}\geq 0
\] and \[
{\eta}_{R}\geq 0
\], representing the minimal amount of money an agent is willing to receive. This allows us a simple control for testing how DoM interacts with utility preferences. In addition, thresholds serve as simple social orientation functions – those with higher thresholds are less likely to make compromises compared to those with low (or zero) thresholds. This serves to introduce diversity in the decision-making process of the agents, represented in principle economic rational agents (reward maximizing agents, who act solely to maximize their utility and do not gain utility from other sources such as manipulation of others, social influence etc.). Other social orientation functions are expected to yield a different behaviour. For example, the Fehr-Schmidt utility (Fehr & Schmidt, 1999) adds to the agent’s utility gain (loss) from inequality aversion. We keep this option for future research.

Formally, the utilities of agents with thresholds *η_s_, η_R_* are:


1 \[
u_{S}^{t}\left({{\eta}_{S}}, a_{S}^{t}, a_{R}^{t}\right)=\left(1-a_{S}^{t}-{{\eta}_{S}}\right)\text{*}a_{R}^{t}
\]

2 \[
u_{R}^{t}\left({{\eta}_{R}}, a_{S}^{t}, a_{R}^{t}\right)=\left(a_{S}^{t}-{{\eta}_{R}}\right)\text{*}a_{R}^{t}
\]

Both agents seek to maximize their discounted long-term reward: \[
{\sum}_{t=1}^{T}u_{i}^{t}{{e}^{\left(t-1\right)\log \left(\gamma \right)}}
\], with a discount parameter \[
\gamma >0
\], here set to \[
\gamma =0.99
\].

Each agent (*i*) uses its DoM level (*k*) to compute the Q-values, \[
{{Q}_{i=k}}\left(a_{i}^{t}|{{h}^{t-1}}, {{\theta}_{i}}\right)
\], which are used for action sampling (policy), *π*. We assume that both parties play a SoftMax policy, with a known temperature \[
\mathcal{T}
\]:


3 \[
P_{i=k}^{t}\left(a_{i}^{t}\vert{{h}^{t-1}}, {{\theta}_{i}}\right)\propto \exp \frac{{{Q}_{i=k}}\left(a_{i}^{t}|{{h}^{t-1}}, {{\theta}_{i}}\right)}{\mathcal{T}}
\]

The action’s Q-value is computed as a function of the history and the agent’s DoM level as described next.

We model the agents using the IPOMDP framework (Gmytrasiewicz & Doshi, 2005). This framework augments the POMDP model to account for modelling others. These models, denoted by *θ*, include all aspects of the other agent’s decision-making characteristics and beliefs. In this task, these aspects include the other agent’s threshold, but it may also include the other agent’s beliefs, including the beliefs of others about the self (i.e., nested beliefs). The level of recursion defines the agent’s DoM level. In this work, we consider an iterated DoM level (Hula, Montague, & Dayan, 2015b) – senders and receivers have odd- and even-numbered DoM respectively.

At the bottom of the hierarchy are DoM(–1) (sub-intentional) agents. DoM(–1) agents are characterized by lacking an opponent model (belief about the other) and are typically considered to be model-free RL agents. In this task, we consider *random* and the *threshold* DoM(–1) senders. The random sender makes offers uniformly random and does not adapt its behaviour to the receiver’s response. We include this sender to examine and test strategies used to exploit the possible existence of a random other. This is both useful to examine when an agent may use the presence of a random other to their advantage (acting as a random agent to instil a sense of powerlessness in their opponent) and thus when an agent’s applied strategy may mistake randomness for intentional policy.

The threshold DoM(–1) senders follow a reactive and myopic policy. If their current offer is accepted, they will offer less in the following iteration as they infer this acceptance as a sign that the offer was “too generous”. On the other hand, if the offer is rejected, they will increase the next offer. Formally, these agents maintain a lower and upper bound representing the range of offers to consider:


4 \[
{{L}^{t}}={{L}^{t-1}}\cdot a_{R}^{t-1}+a_{S}^{t-1}\cdot \left(1-a_{R}^{t-1}\right)
\]

5 \[
{{U}^{t}}={{U}^{t-1}}\cdot \left(1-a_{R}^{t-1}\right)+a_{S}^{t-1}\cdot \left(a_{R}^{t-1}\right)
\]

with \[
{{L}^{0}}=0
\] and \[
{{U}^{0}}=1
\]. In turn, these senders’ Q-values are simply the utility from every action in the range \[
a_{S}^{t}\in \left[{{L}^{t}}, {{U}^{t}}\right]
\]:


6 \[
Q_{S=-1}^{t}\left(a_{S}^{t}; {{\eta}_{S}}\right)=u_{S}^{t}\left(a_{S}^{t}, {{\eta}_{S}}\right)
\]

The DoM(0) receiver models the sender as a DoM(–1) sender. In turn, it forms a belief about the type of the sender – either a random or a threshold sender: \[
{{\hat{\theta}}_{S=-1}}\in \left\{Random, 0.1, 0.5\right\}
\]. These beliefs are updated using IRL (Ng & Russell, 2000). Upon observing an offer \[
a_{S}^{t}
\], the DoM(0) receiver computes the likelihood of the offer for each possible sender type and re-weights them with current beliefs:


7 \[
b_{R=0}^{t}\left({{\hat{\theta}}_{S=-1}}\right)=p_{R=0}^{t}\left({{\hat{\theta}}_{S=-1}}\vert{{h}^{t-1}}, a_{S}^{t}\right)\propto P_{S=-1}^{t}\left(a_{S}^{t}\vert{{h}^{t-1}}, {{\hat{\theta}}_{S=-1}}\right)b_{R=0}^{t-1}\left({{\hat{\theta}}_{S=-1}}\right)
\]

where, \[
P_{S=-1}^{t}\left(a_{S}^{t}|{{h}^{t-1}}, \hat{\theta}\right)
\] is computed using the DoM(0) receiver’s nested DoM(–1) sender model. We assume that the prior beliefs are both common knowledge and flat, making the updated belief common knowledge, as it is a deterministic function of history, and the actions are fully observed. The DoM(0) receiver’s Q-values are a combination of its immediate utility and the discounted expected utility, given that it played \[
a_{R}^{t}
\]:


8 \[
\begin{array}{*{35}{l}} Q_{R=0}^{t}\left(a_{R}^{t};{{\eta}_{R}}, b_{R=0}^{t}\left({{\theta}_{S=-1}}\right)\right)= \\
{E}_{a_{S}^{t+1}\sim \pi_{S=-1}^{\ast}} \left[u_{R}^{t}\left(a_{S}^{t},{{\eta}{R}}\right) \cdot a_{R}^{t}+\displaystyle{\mathop{\gamma\max}_{a_{R}^{t+1}}} \left\{Q_{R=0}^{t+1}\left(a_{R}^{t+1}; {{\eta}_{R}}, b_{R=0}^{t+1}\left({{\theta}_{S=-1}}\right)\right)\right\}\right]
\end{array}
\]

where \[
{{E}_{a_{S}^{t+1}\sim \pi_{S=-1}^{\text{*}}}}
\] is the expected future offer, weighted by the current belief.

Interacting with the simple DoM(–1) sender, these agents solve the optimal policy computation using the ExpectiMax algorithm (Hutter, 2004). This planning algorithm computes the Q-value when playing against a stochastic adversary, by averaging over its expected actions.

Playing with the DoM(0) receiver in mind, the DoM(1) sender includes beliefs about the receiver’s threshold and recursively the receiver’s beliefs about the sender’s type. Due to the known priors and full observability, these nested beliefs are known to the DoM(1), but we specify them here for illustrative purposes:


9 \[
\begin{array}{*{35}{l}} b_{S=1}^{t}\left({{{\hat{\theta}}}_{R=0}},b_{R=0}^{t-1}\left({{{\hat{\theta}}}_{S=-1}}\right)\right)= \\ p_{S=1}^{t}\left({{{\hat{\theta}}}_{R=0}}, b_{R=0}^{t-1}|{{h}^{t-1}}\right)\propto P_{R=0}^{t}\left(a_{R}^{t-1}|{{h}^{t-2}}, a_{S}^{t-1}, {{{\hat{\theta}}}_{R=0}}, b_{R=0}^{t-1}\left({{{\hat{\theta}}}_{S=-1}}\right)\right)b_{S=1}^{t-1}\left({{{\hat{\theta}}}_{R=0}}, b_{R=0}^{t-2}\left({{{\hat{\theta}}}_{S=-1}}\right)\right) \\\end{array}
\]

The DoM(1) Q-values follow the same structure as the DoM(0) Q-values (Equation 8), where the expectation includes the updated beliefs of the DoM(0) receiver upon observing the offer:


10 \[
\begin{array}{*{35}{l}} Q_{S=1}^{t}\left(a_{S}^{t};{{\eta}_{S}},b_{S=1}^{t}\left({{{\hat{\theta}}}_{R=0}},b_{R=0}^{t-1}\right)\right)= \\ {{E}_{a_{R}^{t}\sim\pi_{R=0}^{\text{*}}}}\left[u_{S}^{t}\left(a_{S}^{t},{{\eta}_{S}}\right)\cdot a_{R}^{t}+\displaystyle{\mathop{\gamma\max}_{a_{S}^{t+1}}}\left\{Q_{S=1}^{t+1}\left(a_{S}^{t+1};{{\eta}_{S}},b_{S=1}^{t+1}\left({{{\hat{\theta}}}_{R=0}},b_{R=0}^{t}\right)\right)\right\}\right] \end{array}
\]

Much like the DoM(0) receiver, the DoM(1) sender also uses internal simulation to infer how its actions will affect the receiver. However, while the DoM(0) receiver can manipulate the bounds of the DoM(–1) sender, the DoM(1) sender has a representation of this, and thus can anticipate and predict the beliefs of the DoM(0) receiver to their favour.

We also consider a DoM(2) receiver. This agent models the sender as DoM(1), including all DoM(1) nested beliefs. These nested beliefs are the beliefs the DoM(1) sender ascribes to the presumed DoM(0) receiver as mentioned above. Notably, these receivers also consider the random sender in their model, given that the DoM(1) agent uses the existence of a random agent as a mechanism to deceive the DoM(0). The belief update and Q-values computation follow the same formulation as in Equations (9, 10).

The DoM(1) and DoM(2) agents compute their Q-values using the IPOMCP planning algorithm (Hula et al., 2015b), an extension of the POMCP algorithm to IPOMDP. Using their nested opponent model, these agents plan how to manipulate the policy of the DoM(0) receiver. A summary of the agents properties is presented in Table 1.

**Table 1:** Summary of DoM levels and behaviours. Here we refer to ‘self’ to mean the subject of the belief, and ‘other’ to mean the partner.


	DoM(–1)	DoM(0)	DoM(1)	DoM(2)

DoM characterisation	None	Infer direct action about the other irrespective of the self	Consider others’ beliefs about the self	Consider other’ beliefs about the self thinking about the other

Formalism	None	\[ {{b}_{k=\text{0}}}\left(\theta \right)=P\left({{\eta}_{k=-1}}\right) \]	\[ {{b}_{k=1}}\left(\theta \right)=P\left({{\eta}_{k=0}}\right)\times {{b}_{k=0}}\left(\theta \right) \]	\[ {{b}_{k=2}}\left(\theta \right)=P\left({{\eta}_{k=1}}\right)\times {{b}_{k=1}}\left(\theta \right) \]

Strategic behaviour	Reactive	Identification of the other based on their history of actions	Belief manipulation based on an other’s model (k–1) of the self	Skepticism, Counter-deceptive knowledge that a DoM(1) agent will try and deceive

Task behaviour	Responds to action based on a fixed policy	Adapted behaviour to observations given the identified opponent type	Selects actions to manipulate beliefs given the known world model of a DoM(0) agent	Identify deceptive behaviour and counter-deceive DoM(1)

Vulnerability	DoM(0) manipulation	DoM(1) manipulation	DoM(2) counter-deceptive manipulation	Overmentalizing about DoM(0) and DoM(–1)


In this work we set the experiment parameters to be *T* = 12 and two sender thresholds: \[
{{\eta}_{S}}\in \left\{0.1, 0.5\right\}
\] and two receiver thresholds: \[
{{\eta}_{R}}\in \left\{0.0, 0.35\right\}
\]. In addition, the SoftMax temperature is set to \[
\mathcal{T}=0.01
\]. To ensure our model is robust to initialisations we conducted each DoM pairing for each type of agent (e.g. DoM(–1) random sender vs. DoM(0) receiver with *η_R_* = 0.35, DoM(–1) random sender vs. DoM(0) receiver with *η_R_* = 0.0, etc) with 20 random seeds and then averaged the behavioural outcomes for each dyad for interpretation and visualisation. The combination of these thresholds yields asymmetric outcomes. For example, receivers with higher threshold *η_R_* = 0.35 will act more aggressively (rejecting more offers) vs. their zero threshold counterpart. In turn, the lower threshold sender *η_S_* = 0.1 is likely to “compromise” and improve its offers in response, vs. the higher threshold sender. Other thresholds yield similar patterns, if they satisfy \[
{{\eta}_{S}}+{{\eta}_{R}}<1
\]. The game duration was set to allow agents sufficient time to accumulate enough evidence (likelihood) to detect the type of opponent they interact with. Previous pilot work (Alon, Schulz, Dayan, & Barnby, 2023) used shorter duration, different sender threshold, higher SoftMax temperature and no receiver threshold and yielded similar results. We present the results from this pilot work in the appendix. We also experimented with higher SoftMax temperature \[
\mathcal{T}=1.0
\] to evaluate the effect of high temp on detectability and consequent behaviour. These results are discussed later.

## Results

We begin by analyzing the cases where the agents’ DoM levels are typically, or adaptively matched, *i.e.*, where one agent has DoM(*k* + 1) and the other DoM(*k*).^1^ These simulations establish a baseline of typical strategic behaviour stemming from the higher DoM agent’s ability to manipulate the beliefs of its counterpart through actions.

First, since higher DoM agents (such as DoM(1)) model the beliefs of lower DoM agents (such as DoM(0)) to their strategic benefit, a DoM(>1) agent can confuse naive behaviour as arising instead from a DoM(1). We account for this by analyzing the counter-deceptive reasoning applied by DoM(2) agents and show how this sophisticated strategy can be a blessing when matched with DoM(1) agents, and be disadvantageous when matched with simpler agents.

Second, DoM(2) agents believe themselves to be interacting with DoM(1) partners, and can believe that DoM(1) partners are strategically impervious to the responses of what they assume to be their DoM(0) opponents. Thus, DoM(2) agents can exhibit a form of helplessness when playing DoM(–1), when in fact they would be perfectly capable of exploiting them appropriately, using the nested model of DoM(–1) agents and planning through their behaviour. We illustrate this by simulating the DoM(2) receiver and the DoM(–1) sender. Due to the strictness of opponent reasoning of the cognitive hierarchy, the DoM(2) models its counterpart as DoM(1), misinterpreting the behaviour of the DoM(–1) sender. From these simple reward-maximising mechanisms enacted in a competitive, interactive context, we find that sophisticated opponents are vulnerable to over-mentalizing and subsequent loss of reward.

### Baseline behaviour

Theory of Mind (ToM) is used for both inference and planning. For example, when the DoM(0) receiver observes an offer by the sender, its belief update allows it to identify the type of sender by inverting the offer to infer the sender’s characteristics. Here, the sender is assumed by the receiver to have a DoM(–1) policy: it is unable to mentalize about the receiver. The DoM(0) receiver can then use its model of the sender to simulate how each sender type would respond to the receiver’s action, weighing the optimal response according to its beliefs. Thus, it can manipulate the sender’s behaviour to its benefit within the bounds of any inherent irreducible uncertainty.

#### DoM(–1) sender and DoM(0) Receiver: Näive utility calculus

**Figure 2:**
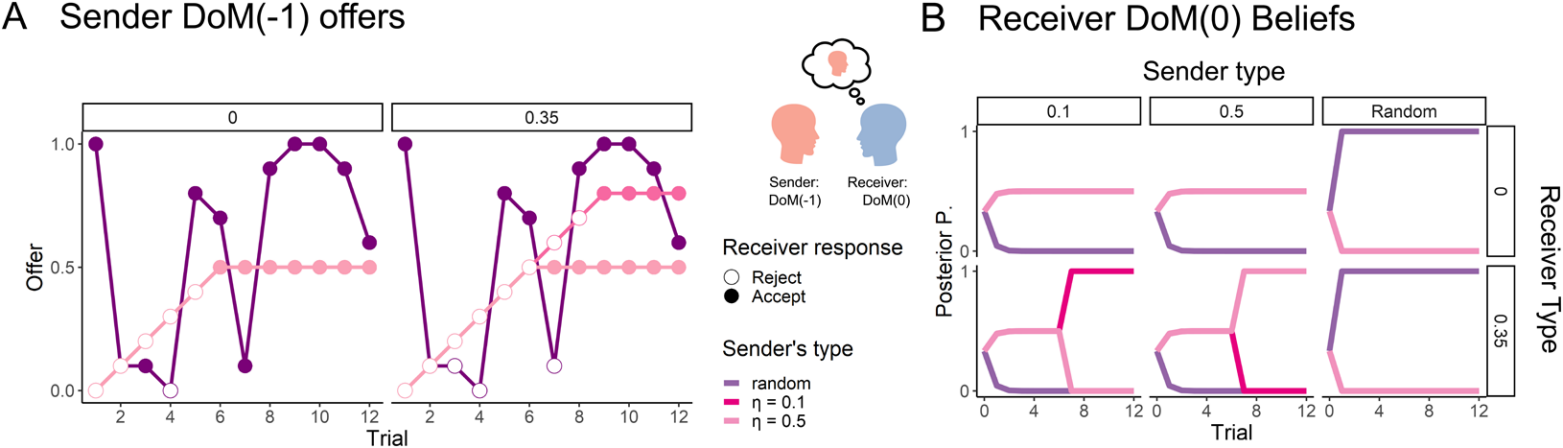
**Illustration of DoM(0) IRL: (A, B)** In interacting with the DoM(–1) sender (A), the DoM(0) receiver makes inferences about the sender’s type (B). Notably, the first offer is usually sufficient to tell the random sender from the threshold senders. When the receiver’s belief favours the threshold sender, the receiver manipulates the sender by rejecting the offers until a desired offer is met, according to the receiver’s threshold. Both DoM(–1) threshold agents are *reactive* – that is, they respond to the behaviour of others. Hence they react similarly to the strategic behaviour of the DoM(0) until their “willingness” to bounded is limited by their threshold (after 6 trials) – the main difference between their behaviour is the maximal offer they are willing to make. The thresholds of the agents determine the range of possible agreement – agents with higher thresholds are less willing to “compromise”. For example, agents (both receiver and sender) with higher thresholds need a more egalitarian split of the endowment compared to those with low thresholds. Note: Posterior *P*(*θ*) means the posterior distribution of the inferring agent after observing the actions of the other agent. *P*(*θ*) = 0 means that the inferring agent’s belief places zero probability that the observed agents has type *θ* and *P*(*θ*) = 1 means that the inferring agent is certain that the observed agent has type *θ* (when lines overlap the behaviour of the DoM(–1) sender or the updated beliefs of the DoM(0) are the same for both thresholds).

Following the properties of hierarchical mentalizing, we begin with the first dyad of adaptively aligned DoM – a DoM(–1) sender interacting with a DoM(0) receiver. The DoM(0) inference about the DoM(–1) type is displayed in Figure 2(B). Crucially, in this example, the first offer is enough to parse whether the partner is a random sender or a threshold sender (since the offer is so high). After making this distinction, the receiver adapts its policy. If the beliefs support the threshold sender, the optimal policy is to reject the offers, pushing the lower bound upward until a desired level is met. Figure 2(A) shows this manipulation as a function of the receiver’s threshold – the zero threshold receiver’s acceptable offer is 0.5 (which is the maximal offer the DoM(–1) sender with *η* = 0.5 is willing to make), while the 0.35 threshold receiver is “demanding” a higher offer to maximize its long term cumulative reward. On the other hand, if the DoM(0) receiver believes it is facing the random sender, it accepts any offer that satisfies its threshold, as the random agent cannot be manipulated. This is behaviour is appropriate given the context.

#### DoM(1) sender and DoM(0) Receiver: Deception through induced false beliefs

**Figure 3:**
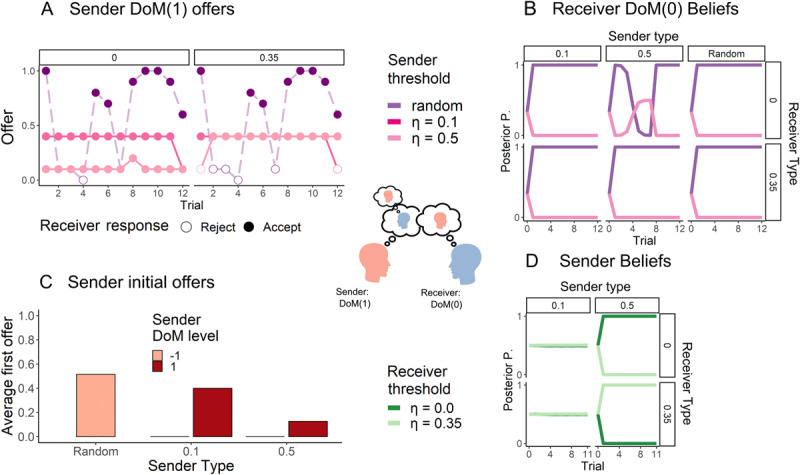
**Dynamics of the DoM(1) manipulation:** The DoM(1) offers are strategically aimed at shifting the DoM(0) belief in favour of the random sender hypothesis. This strategy naturally arises out of simple reward-maximising agents in a mixed motive setting. **(A)** Illustrative offers sent by different types of senders within a single seed. The sender’s initial offer “mimics” that of the random sender, before subsequently defecting. **(B)**, The DoM(0) false beliefs about the identity of the DoM(1) agent due to the strategy the DoM(1) player uses. The DoM(1) offers are deliberately high, to be classified by the DoM(0) beliefs as coming from a random sender. While the DoM(–1) sender’s first offer is 0.0, the DoM(1) sends between 0.1 and 0.4. **(C)** The DoM(1) sender’s deception is characterized by making a relatively high first offer. This offer is highly atypical for a DoM(–1) threshold sender. **(D)** using the same IRL concept, the DoM(1) makes inferences about the DoM(0) receiver’s type from its responses. Note: Posterior P. means the posterior distribution of the inferring agent after observing the actions of the other agent. *P* = 0 means that the inferring agent’s belief places zero probability that the observed agent has type *θ* and *P* = 1 means that the inferring agent is certain that the observed agent has type *θ* (when lines overlap the behaviour of the DoM(1) sender or the updated beliefs of the DoM(0) are the same for both thresholds).

The DoM(1) sender uses its DoM(0) nested model to compute what should be an optimal policy. It emulates the DoM(0) inference process and consequently predicts the DoM(0) policy. Given the policies depicted in Figure 2, the DoM(1) sender’s policy is to take actions consistent with a random DoM(–1) agent, causing the DoM(0) to accept any offer (respecting the receiver’s threshold). This set of random-like actions arises through the ability of the DoM(1) to model fully the expectations, beliefs, and mentalizing capacity of the DoM(0) (Kopp, Korb, & Mills, 2018); Acting in the same way as a random sender utilizes the DoM(1)’s ability to make inferences about the DoM(0)’s IRL process. The belief manipulation allows the DoM(1) sender to avoid the strategic rejection policy of the DoM(0) receiver, as from the DoM(0) perspective, the offers are highly unlikely for a threshold sender, illustrated in Figure 3(C). Given the low SoftMax temperature, the DoM(1) correctly infers that the DoM(0) receiver would infer that any offer other than 0.0 is highly likely to come from the random sender, as the threshold DoM(–1) senders are expected to start by offering nothing.

Once false beliefs have developed in the DoM(0) (approximated also by the DoM(1)), the DoM(1) sender’s policy is to repeatedly send the bare minimal offer (presented in 3(A)), to extract reward at the expense of the DoM(0). As the likelihood of a flat trajectory of offers is the same as the likelihood of any other trajectory generated by a random sender, the DoM(0) receiver is unable to tell the true random from the fake one as depicted in Figure 3(B).

#### DoM(1) sender and DoM(2) Receiver: Defying deception with deception

**Figure 4:**
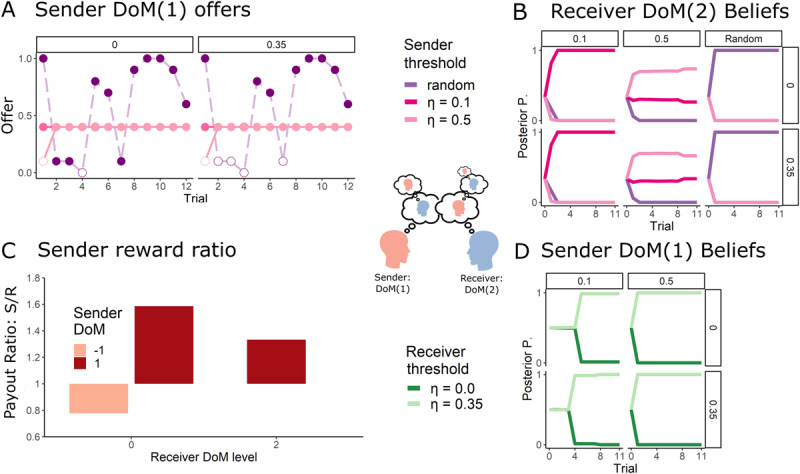
**Dynamics of the DoM(2) counter-manipulation: (A)** The DoM(2) with low threshold, masquerading as the high threshold receiver, rejects low offers. This encourages the DoM(1) sender with a high threshold to improve its offers, while having little effect on the already “generous” *η* = 0.1 DoM(1) sender. **(B)**, the DoM(2) receiver correctly reads the DoM(1) sender’s strategy, while manipulating the latter’s beliefs **(D)** As depicted in **(D)**, this causes the DoM(1) to develop false beliefs about the identity of the DoM(2) as they are unable to model them appropriately. **(C)** Typically, the agent with the higher DoM gains a higher reward than the lower DoM agent. The y-axis measures the ratio between the receiver and sender’s total reward. Due to the asymmetric nature of the IUG, the DoM(2) receiver superiority is manifested in its ability to lower the DoM(1) sender advantage. Note: Posterior P. means the posterior distribution of the inferring agent after observing the actions of the other agent. *P* = 0 means that the inferring agent’s belief places zero probability that the observed agent has type *θ* and *P* = 1 means that the inferring agent is certain that the observed agent has type *θ* (when lines overlap the behaviour of the DoM(1) sender are the same for both thresholds).

The DoM(2) can simulate the policy of the DoM(1), and all nested models of the DoM(1) sender. The DoM(2) can anticipate that “random” offers may arise from a sophisticated DoM(1), and thus can react accordingly (Figure 4). Applying the same belief manipulation principles as the DoM(1), the DoM(2) acts in a way that causes the DoM(1) to falsely believe that it is matched with the higher *η* = 0.35 DoM(0) receiver, thus pressuring the sender to improve its offers in the case of the lower threshold DoM(2) receiver. This yields a higher reward compared to the limited-opponent modelling DoM(0) receiver. Notably, due to the built-in advantage of the sender in this task (the sender has to offer at most 0.4), the DoM(2) enjoys a decrease in the sender to receiver reward ratio.

We conclude that when appropriately matched, being a DoM(*k* + 1) matched with a DoM(*k*) partner is beneficial. These findings reinforce previous work highlighting the advantages of higher DoM in mixed-motive games. Figure 4(C) illustrates this supremacy – the total reward ratio is always in favour of the higher DoM agent.

We now examine the behavioural phenotype of a high DoM(2) receiver matched with a very simplistic DoM(–1) sender; in this case, the DoM(2) receiver is using a very sophisticated strategy for very simple sender.

### Skepticism and paranoia in DoM(2)

As we will see, DoM is a double-edged sword. A mismatched DoM agent may misinterpret the actions of their partner, misinterpreting simplistic behaviour as the product of Machiavellian sophistication. Here, predicting that a DoM(1) sender would act like a random sender to deploy deception, the DoM(2) receiver is susceptible to interpreting random behaviour as having been generated by the DoM(1) sender, even when this is not the ground truth. This leads to delayed detection of a true random sender, as more evidence of “random” behaviour is required to confirm that the sender is genuinely random. The very possibility that random-like behaviour may be used as deception has the effect that it takes, on average, 5 trials for the DoM(2) receiver to converge to the true-random type compared to the 2 trials it takes on average for the DoM(0).

Delayed random identification has a limited effect on the DoM(2) reward when in the presence of a DoM(1). Nevertheless in the presence of a DoM(–1), the DoM(2) incurs more severe reward loss.

**Figure 5:**
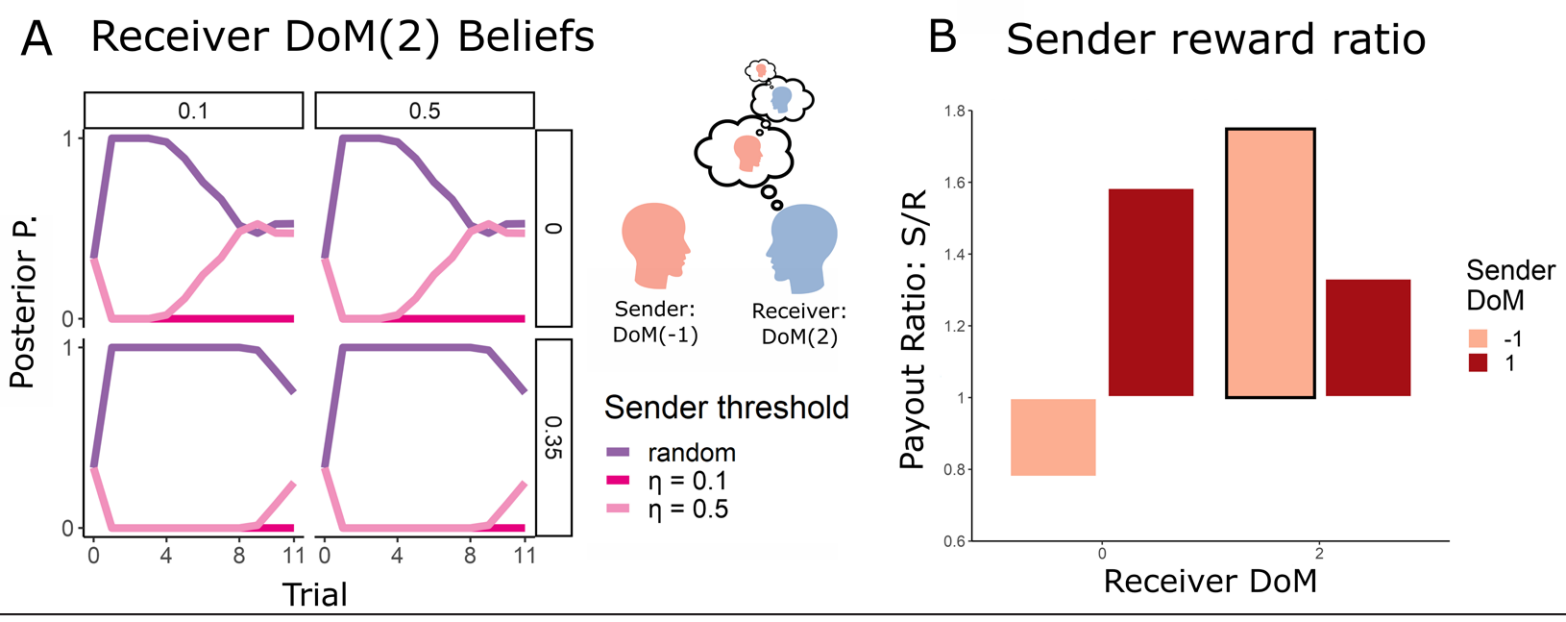
**Effects of maladaptive DoM: (A)** The DoM(2) has developed false beliefs about their unsophisticated DoM(–1) partner. This is because offers of the DoM(–1) sender lie outside the DoM(2) opponent model and are viewed as coming from a random sender. **(B)** In turn, the receiver’s docile policy means that they are willing to accept any offer, yielding them a low reward. Note: Posterior P. means the posterior distribution of the inferring agent after observing the actions of the other agent. *P* = 0 means that the inferring agent’s belief places zero probability that the observed agent has type *θ* and *P* = 1 means that the inferring agent is certain that the observed agent has type *θ*.

In cases of DoM(2) vs. DoM(–1), the over-attribution of sophisticated strategic intention to observations, typical of the DoM(1), means that the DoM(2) receiver fails to model the DoM(–1) sender. On the one hand, as mentioned, true randomness is interpreted as arising from a DoM(1). But there is also a double-bind: as the low offers of the threshold DoM(–1) senders are atypical for the random-pretending DoM(1), the DoM(2) receiver also interprets any non-random DoM(–1) actions as a sign of random behaviour as depicted in Figure 5(A).

Given that the best response for a receiver in the face of a truly random sender is to accept anything above one’s threshold, the trapped DoM(2) receiver accepts most of the threshold DoM(–1) sender offers. However, the simplistic DoM(–1) senders will improve their offers only if rejected, otherwise, they continue to make the same low offer. The detriment to the receiver is evident in Figure 5(B). In effect, the DoM(2) never acts to cause the DoM(–1) to show itself to be able to be changed, and so never encounters evidence against its own beliefs. The DoM(2) believes one interpretation of events and thereby avoids any chance of encountering disconfirmatory evidence.

While the DoM(2) falls for the same deception applied by the DoM(1) sender against the DoM(0) receiver, the causal mechanism between the two differ. In the case of a DoM(–1) sender, the DoM(2) is a victim of its sophistication, and the incorrectly attributed sophistication of its partner, and not the victim of a truly savvy opponent.

### Influence of high environmental noise

Our results highlight the attribution of *intention* to random behaviour in sophisticated agents. The DoM(2) maps the random-like behaviour of the DoM(1) as an intention to deceive. At the same time, the DoM(0), lacking the capacity to simulate such an opponent, attributes the same behaviour to a random sender. However, this detectability of intention is plausible due to the sender’s close mapping of their beliefs to their actions – we chose to keep the decision temperature low. This means that from the DoM(0)’s perspective, the DoM(–1) threshold senders’ behaviour is quite predictable, hence easy to invert. The question arises: Can agents still infer intentions if the environment is stochastic and noisy? To foreshadow, we show that noise is necessary but insufficient to induce the same false beliefs of malice in the DoM(2) receiver.

**Figure 6:**
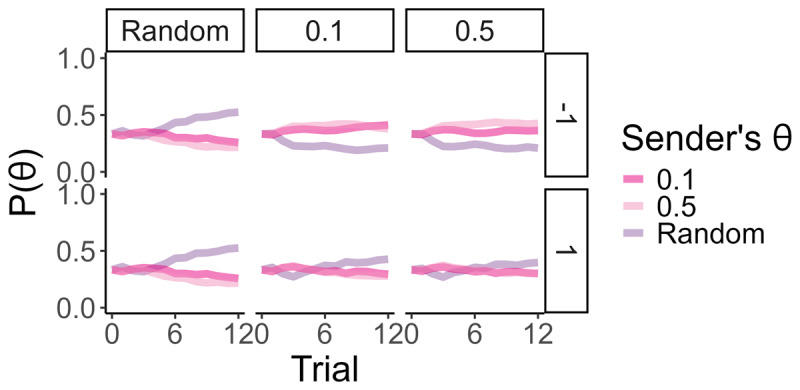
**DoM(0) belief update in high SoftMax temperature environment.** We depict the updated DoM(0) beliefs against senders with different DoM level (row indicate sender’s DoM level, column indicate sender’s type) averaged across 20 different simulations. Due to the noisy behaviour of the senders, the DoM(0) finds it hard to identify the sender’s correct beliefs from its actions, even when it interacts with an adaptively matched sender (DoM(–1), top row). When interacting with the higher DoM sender, the receiver is still deceived, but with a lower certainty.

To examine this issue, we simulated the dyads with a high SoftMax temperature \[
\mathcal{T}=1.0
\]. In this case, the non-random agents’ behaviour is more stochastic, making Bayesian IRL hard as the mapping between beliefs and actions is tenuous. We begin with the DoM(0) baseline. In the high-temperature environment, the receiver is barely able to detect the correct type of the sender, as evident in Figure 6 (Top row). When interacting with the DoM(1), the DoM(0) is again still “fooled by randomness”, inferring the DoM(1) as a random sender, but with a lower degree of certainty, as depicted in Figure 6 (Bottom row).

**Figure 7:**
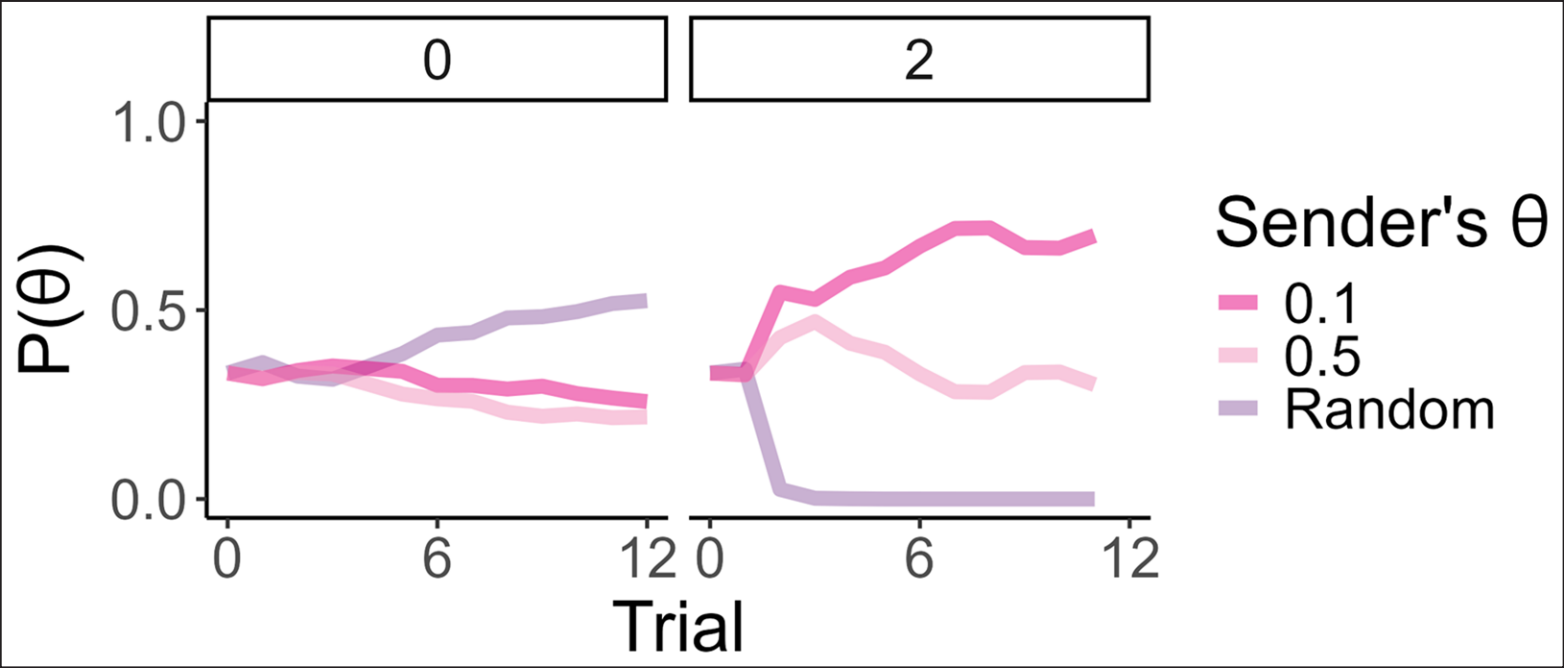
**Comparison of updated beliefs against a random DoM(1) sender in high SoftMax environment.** We depict the updated receiver beliefs interacting with a random sender averaged across 20 different simulations. The DoM(0) attributes the random behaviour of the random sender(left panel). On the other hand, the DoM(2) receiver attributes the random behaviour to a benign, high-temperature DoM(1) sender.

The DoM(2) inference yields different results, presented in Figure 7. To surmise, DoM(2) receivers view the stochastic actions as unintentional. By inverting the offers of a random sender through the lens of the DoM(1), the DoM(2) belief update takes into account the nested DoM(0) beliefs, and thus the poor identification of the sender. Consequently, the DoM(2) falsely attributes the stochastic behaviour to a noisy DoM(1) threshold sender as evident in 7. This misidentification is caused by the DoM(2) receiver inferring offers as stemming from a low-threshold sender – the offers are “too high” to be counted as being generated by a high-threshold sender, even in a stochastic environment.

## Discussion

We show that hierarchical mentalizing is a double-edged sword. We analysed pairs of RL agents endowed with ToM at different depths of mentalizing in a mixed-motive game. When agents correctly model their opponent’s degree of sophistication, they can protect themselves, acting appropriately against deceptive partners. These simulations are aligned with the hypothesis that ToM has evolved out of the need to survive and succeed in complex mixed-motive environments (Lyons, Caldwell, & Shultz, 2010; Whiten & Byrne, 1988; Qi & Vul, 2020). On the other hand, we also show how high DoM can be maladaptive when miscalibrated: Agents thinking three steps into the cognitive hierarchy become sceptical against even random behaviour and are trapped in an over- or hyper-mentalized policy, believing they are matched with a sophisticated other that will try to deceive. Importantly, we show that both ambiguous behaviour and intentionality are necessary and sufficient to explain maladaptive overmentalizing – the former in the absence of the latter is explained away as unintentional noise. This phenomenon, generated purely from two simple reward-maximising agents in an interactive context, makes for a plausible explanation for the generation and maintenance of psychopathological states, such as paranoia, where misperceiving others’ negative intentions is a central feature and important source of disability.

Our work highlights how maladaptive DoM is a product of the agent’s own beliefs, its environmental context, and the internal representation of an other. This is consistent with prior observations (Simon, 1990; Bhui, Lai, & Gershman, 2021; Huys, Guitart-Masip, Dolan, & Dayan, 2015), and is relevant for the maladaptive behaviour of machines (E. Schulz & Dayan, 2020). It also shows how complex phenomena like scepticism can arise even from optimal Bayesian inference (Bhui & Gershman, 2020; Alon, Schulz, Rosenschein, & Dayan, 2023) and how optimal Bayesian inference can go awry given confusion about a decision problem or an unfortunate environment (Huys, Guitart-Masip, Dolan, & Dayan, 2015).

The over-attribution of negative social intentions is a central feature in paranoid delusions and borderline personality disorder (Buck et a., 2023) and indeed hyper-mentalizing has been identified as an important transdiagnostic feature in psychopathology more broadly (McLaren et al., 2022; Sharp et al., 2011). Our work offers a computational model of these phenomena, formalising a theory of how hierarchical, recursive social cognition gone awry may explain the emergence of paranoia, which may be maintained in purely reward-maximising, interactive agents with minor miscalibrations. This cognitive mechanism may play a crucial role in the formation of persecutory delusions, along with inflexible priors about interaction partners (Barnby, Raihani, & Dayan, 2022; Diaconescu, Wellstein, Kasper, Mathys, & Stephan, 2020; Wellstein et al., 2020), noisy mental models of others (Adams, Vincent, Benrimoh, Friston, & Parr, 2022; Barnby, Mehta, & Moutoussis, 2022), social hypersensitivity (Henco et al., 2020), and biased social values (Kazinka, Kwashie, Pratt, Vilares, & MacDonald III, 2023). Of note, our models also offer a glimpse into the emergence of biases against disconfirmatory evidence (Freeman, 2016), given that hypermentalized receivers in our simulations form their fixed beliefs quickly, and thus fail to expose themselves to actions which may cause belief reorganisation.

Crucially, the DoM(2) discerns between intended and unintended decision noise. The DoM(2) infers stochasticity as a sign of intended strategy only in the low SoftMax environment – it attributed random behaviour as an intentional choice by the strategic DoM(1) partner. In contrast, in the high-temp environment, the DoM(2) infers the stochastic behaviour as an unintentional noisy execution of the DoM(1)’s policy. Our model explains why noisy environmental expectations have typically been associated with persecutory beliefs in reward tasks (e.g. Hauke et al., 2024) and in traditional vignette tasks (e.g. Kinderman, Dunbar, & Bentall, 1998), but highlights that this in of itself is insufficient to explain the intentionality of attributions of intentional harm. The addition of our high-SoftMax simulations shows a distinction: 1. noise is necessary, and 2. it must be viewed as intentional – a characteristic only possible in DoM(2) agents.

The results of the simulations pave the path for human experiments. Paradigms testing this theory directly should use the IUG in their designs, with computerized partners (senders) calibrated across differing levels of DoM from k = –1 to k = 2. Participants will play different partners in a within-subject design over multiple games, each game consisting of at least 12 trials as per our simulations. Given parallel work in social interaction and hierarchical cognition (Bürgi, Aydogan, Konovalov, & Ruff, 2024; Qi & Vul, 2020) we would expect most participants to be successful in adapting their DoM to the complexity of their partner. Nevertheless, we would expect some deviations: 1. High trait paranoia will be significantly associated with mismatched DoM, such that simple partners (DoM(k = –1) or DoM(k = 0) are met with relatively high DoM (DoM(k = 1) or DoM(k = 2), respectively) by the participant (ascertained through model fitting). Testing this paradigm in patients with persecutory delusions will be a core aspiration. We would expect that patients with persecutory delusions will make the same errors as those with high trait paranoia in the general population (demonstrating overly deep DoM), although do so with greater frequency and rigidity (taking longer, if at all, to match their partner’s DoM appropriately). We would also expect D2/D3 antagonism to reduce this tendency in both general (Barnby, Bell et al., 2023) and clinical (Adams et al., 2022) populations. Across the board, patients with persecutory delusions and those with high trait paranoia should be equally effective as other participants when matched with senders at DoM(1) who are sophisticated and deceptive. In this case, occupying a higher DoM is adaptive.

Our simulations rely on a well-established game, and relatively simplistic agents to focus on exemplar emergent behaviours explaining the production of false beliefs around the strategic and malicious nature of others, but this naturally introduces limitations. First, we use simple, fixed thresholds to determine the utility type of the sender. Indeed, Fehr-Schmidt (FS) (Fehr & Schmidt, 1999) or FS-like utility functions are typically used to assess rejection in social contexts (e.g. Hula et al., 2018; Kazinka et al., 2023), although we opted to remove this to isolate the effect of DoM. Replacing these egocentric utilities with social orientation utilities, like inequity aversion (Hughes et al., 2018), may yield other non-trivial effects of hypermentalizing.

Second, our model assumes a strict k-level model. This means that an agent’s interpretation of the opponent is bounded to a fixed level of DoM, making the higher DoM agents susceptible to over-mentalization and unable to assume otherwise. One remedy for this problem, which future work may explore, is adopting a mixture model view of the cognitive hierarchy. In this version, suggested by (Camerer et al., 2004), a DoM(*k*) views the world as composed of different levels of DoM levels, ranging from (*k* – 1) to (–1), distributed according to a truncated Poisson distribution. This model may solve the problem of over-mentalization, as the higher DoM agent no longer treats others as having a fixed DoM(*k* – 1) but rather has multiple (unknown) DoM levels. However, this instantiation comes with an increase in the computational costs and complexity of the inference process.

One future direction for solving fixed over-mentalization is to make the DoM level an intentional, adaptive parameter. For example, after learning a partner is not attempting to deceive, one’s own DoM might reduce to fit the context (although the potential sophistication of the agent remains constant). A potential source and consequence of psychiatric symptoms might be a delay in making this reduction even when the computational and utility costs are high. Again, we predict that those with high vs low paranoia would enter into high DoM states much faster and take longer to reduce to adopting a lower DoM when the environment is less competitive.

Another natural extension of our model may also incorporate sophistication detection: the ability for an agent to recognise when it is up against a more sophisticated partner, even if it cannot change its own DoM. This is relevant in several real-world scenarios and may offer a heuristic ‘cheat’ to the k-level hierarchy rationale. For example, humans, particularly those who are paranoid, can believe that they are being confronted with agents who are smarter than them and whose actions lack a transparent rationale – one can sense a plot is afoot but be unable to fully conceptualise it. Such an extension would allow an agent to make heuristic responses, such as threats to exit a context if they could not out-manoeuvre their partner strategically by increasing their mentalization depth (Hertwig & Engel, 2016; Hula et al., 2018). A necessity of this modification requires a metacognitive understanding of the limitations of one’s social cognition. Such metacognition might also be employed to make other decisions before drastic action, e.g., gathering more information about opponents (L. Schulz, Fleming, & Dayan, 2023).

## Figures and Tables

**Figure 8:**
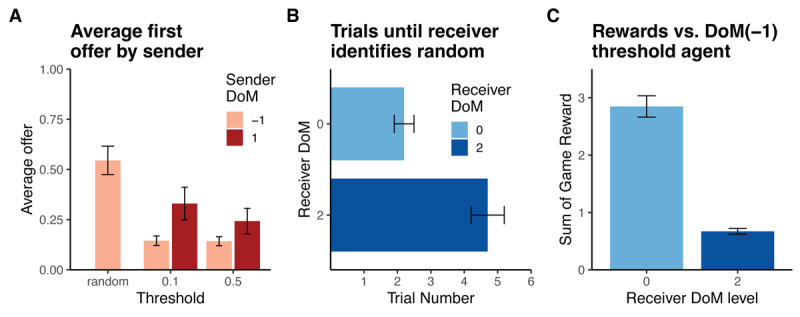
**Results summary:** The rise of deception as well as rational and irrational paranoia is captured by three results: **(A)** Sending high initial offers is a signature of the random agents. In contrast, DoM(–1) senders with thresholds send lower initial offers. This signature is exploited by higher DoM senders which essentially masquerade as random agents by sending higher initial offers and as a result trick the receiver into accepting lower offers later on. **(B)** Sophisticated receivers are aware of this, taking a lot longer to be convinced that they are playing with a random source (We plot the average number of trials until a receiver has reached 99% certainty that it is playing with a random source when it is playing with a random source). **(C)** While this is prudent when the DoM(2) receiver plays with a deceptive DoM(1) sender, it is irrationally paranoid when it plays with DoM(–1) random agent, causing the DoM(2) to sustain losses. Throughout these plots, we show the means and standard errors of the mean.

## Footnotes

Note that in this case the DoM levels are matched and adaptive from the perspective of the DoM(*k* + 1), but this is untrue for the DoM(*k*) who cannot conceive of the DoM(*k* + 1),’s world model.

## Appendix

### Experiments with other parameters

In (Alon, Schulz, Dayan, & Barnby, 2023), the IUG task was simulated with different set of parameters \[
{{\eta}_{S}}\in \left\{0.1, 0.5\right\}
\], \[
{{\eta}_{R}}=0.0
\], \[
\mathcal{T}=0.05
\] and *T* = 10. The main findings are similar to the ones presented in this work and are displayed in Figure 8.

### IRL as ToM inference

As presented above, inference in ToM can be seen as an extension of inverse RL (IRL) (Ng & Russell, 2000). Bayesian IRL (Ramachandran & Amir, 2007; C. Baker et al., 2011) requires an observer to make a Bayesian inference about the utility (reward) function of an agent from a sequence of observed behaviour \[
{{o}^{0:T}}
\]:

11
\[
p\left(u\vert{{o}^{0:T}}\right)\propto P\left({{o}^{0:T}}\vert u\right)p(u)
\]

The DoM(0) inference follows this principle, inferring about the DoM(–1)’s utility function from its behaviour, using a nested model. This observation was made before (Jara-Ettinger, 2019), and framed as the Näive utility calculus (Jara-Ettinger, Gweon, Schulz, & Tenenbaum, 2016). Formally, this inference requires a commonly known behaviour of the DoM(–1) (Equation 5). This behaviour is composed of the DoM(–1) Q-values (Equation 6) and its policy (Equation 3). Plugging into Equation 7 give rise to the IRL process, effectively a posterior distribution over the utility functions, as in Figure 2B.

While following the same principles, namely inverting the behaviour to infer about the mechanism, higher DoM agents inference goes beyond utility inference. In this case, the inference also includes the agent’s beliefs (Equation 9). Notably, if the common prior or action observability assumptions are revoked, the inference process yields a multi-dimensional distribution: \[
p\left(\theta \times b\left(\cdot \right)\right)=p\left(\theta \right)\times p\left(b\left(\cdot \right)\right)
\]. The first component is similar to the utility inference of the DoM(0) agent, while the second one is a distribution over distributions (Hjort, Holmes, Müller, & Walker, 2010). We refer the reader to (Gmytrasiewicz & Doshi, 2005) for a full introduction of belief update in this case.
